# Aetiological role of common respiratory viruses in acute lower respiratory infections in children under five years: A systematic review and meta–analysis

**DOI:** 10.7189/jogh.05.010408

**Published:** 2015-06

**Authors:** Ting Shi, Kenneth McLean, Harry Campbell, Harish Nair

**Affiliations:** 1 Centre for Global Health Research, Usher Institute of Population Health Sciences and Informatics, University of Edinburgh, Edinburgh, Scotland, UK; 2 Centre for Population Health Sciences, Usher Institute of Population Health Sciences and Informatics, University of Edinburgh, Edinburgh, Scotland, UK; 3 Centre for Medical Informatics, Usher Institute of Population Health Sciences and Informatics, University of Edinburgh, Edinburgh, Scotland, UK; 4 Public Health Foundation of India, New Delhi, India; * Joint authors in this position.; † Joint authors in this position.

## Abstract

**Background:**

Acute lower respiratory infection (ALRI) remains a major cause of childhood hospitalization and mortality in young children and the causal attribution of respiratory viruses in the aetiology of ALRI is unclear. We aimed to quantify the absolute effects of these viral exposures.

**Methods:**

We conducted a systematic literature review (across 7 databases) of case–control studies published from 1990 to 2014 which investigated the viral profile of 18592 children under 5 years with and without ALRI. We then computed a pooled odds ratio and virus–specific attributable fraction among the exposed of 8 common viruses – respiratory syncytial virus (RSV), influenza (IFV), parainfluenza (PIV), human metapneumovirus (MPV), adenovirus (AdV), rhinovirus (RV), bocavirus (BoV), and coronavirus (CoV).

**Findings:**

From the 23 studies included, there was strong evidence for causal attribution of RSV (OR 9.79; AFE 90%), IFV (OR 5.10; AFE 80%), PIV (OR 3.37; AFE 70%) and MPV (OR 3.76; AFE 73%), and less strong evidence for RV (OR 1.43; AFE 30%) in young children presenting with ALRI compared to those without respiratory symptoms (asymptomatic) or healthy children. However, there was no significant difference in the detection of AdV, BoV, or CoV in cases and controls.

**Conclusions:**

This review supports RSV, IFV, PIV, MPV and RV as important causes of ALRI in young children, and provides quantitative estimates of the absolute proportion of virus–associated ALRI cases to which a viral cause can be attributed.

Acute lower respiratory infection (ALRI), including pneumonia, remains the leading cause of childhood hospitalization and mortality in young children (under 5 years old) [1], primarily within developing countries [2]. It has been previously estimated that there were 11.9 million and 3.0 million respective episodes of severe and very severe ALRI that contributed to childhood hospitalization globally in 2010 [3]. Furthermore, there were 0.935 million attributable deaths in 2013 [4].

*Streptococcus pneumoniae* and *Haemophilus influenza type b* (Hib) have been established as the principal aetiological agents of pneumonia – together thought to cause over 50% of all severe ALRI cases in developing countries [5]. The childhood vaccination programme against these bacteria [6] is associated with a substantial reduction in morbidity and mortality from ALRI [7]. Continued research is required to further understand the role of other ALRI pathogens, such as viruses.

Respiratory viruses are implicated, either directly, or as synergistic pathogens or co–factors in bacterial superinfections, in up to two thirds of all cases of pneumonia (equating to 80 million cases in young children in 2010) [2,8]. Respiratory syncytial virus (RSV) is the most commonly identified virus in young children with ALRI, contributing to an estimated 33.8 million new cases globally in 2005 [9]. Also, at least 25 other viruses have been associated with ALRI in children, most notably – rhinovirus, influenza, human metapneumovirus (MPV) and parainfluenza viruses (PIV) [8]. However, their aetiological role in ALRI in young children remains uncertain and we are not aware of any systematic reviews currently published that investigate this.

Therefore, it is important to understand these viruses’ contribution to ALRI. We aimed to conduct a systematic review to identify all case–control studies investigating the potential role of respiratory viruses in the aetiology of acute lower respiratory infections in children younger than five years of age.

## METHODS

### Search strategy and selection criteria

We conducted and reported a systematic review according to the PRISMA guidelines. We used tailored strategies to search Medline, Embase, Global Health, LILACS, China National Knowledge Infrastructure (CNKI), Wanfang Data and Chongqing VIP databases (**Online Supplementary Document(Online Supplementary Document)**). We further hand–searched the table of contents of specialist journals – the *Influenza and Other Respiratory Viruses* and *Pediatric Infectious Diseases Journal* – and the reference lists of relevant papers for eligible articles. All searches were limited to between 1 January 1990 and 4 April 2014, and there were no publication status or language restrictions applied.

We included studies that fulfilled our strict eligibility criteria: studies in children younger than five years; studies investigating clinical pneumonia (or lower respiratory infection) as the primary outcome; studies where respiratory specimens were collected and diagnostic test conducted using valid laboratory tests; case–control studies / prospective cohort studies that reported data in both case and control groups; reporting virus–specific proportions separately in both groups; studies published between 1 January 1990 and 4 April 2014 (19 March for Chinese databases). We only included studies where the case definition for ALRI (or clinical pneumonia) was clearly defined and consistently applied.

Two investigators (TS and KM) conducted independent English language literature searches and extracted data using standardised data extraction templates. One investigator (TS) whose first language is Chinese performed the search and data extraction from Chinese language databases (CNKI, Wanfang and CQVIP). Any discordance and/or uncertainties regarding relevance or inclusion were arbitrated by HN or HC.

### Definitions

We used “ALRI” as an equivalent to clinical pneumonia as our case definition, which also included bronchiolitis. This was to recognise this common manifestation in young children with viral ALRI [10], and the limits of the WHO case definition to reliably differentiate these [1]. ALRI was characterized as cough or dyspnoea with age–related tachypnoea, while severe ALRI was defined as those with cough or dyspnoea with indrawing of the lower chest wall [11], or an acute respiratory infection severe enough to necessitate hospitalisation. The control groups were defined as asymptomatic (with no respiratory symptoms), healthy (asymptomatic with no other symptoms) or upper respiratory tract infection (URTI) (with respiratory symptoms).

### Statistical analysis

We standardised the results of all the included studies as odds ratios (ORs) with accompanying 95% confidence intervals (95% CIs), to facilitate interpretation and comparison. We applied a continuity correction of 0.0005 if a virus was detected in one group, but not the other [12]. This allowed calculation of an OR for these instances, and enabled inclusion within subsequent meta–analyses. Furthermore, matched (mOR) and adjusted (aOR) odds ratios were also extracted, where possible. These were used preferentially in subsequent calculations and analyses.

Using STATA (version 11.2), we performed a meta–analysis of virus–specific ORs and reported pooled estimates with corresponding 95% CIs using the random effects model (DerSimonian–Laird method) because these studies do not share common effect size due to methodological heterogeneity [13]. The virus–specific attributable fraction among the exposed (AFE) was used to explore the etiological role of each virus in ALRI patients. This estimates the percentage of (severe) ALRI which can be attributed to each virus, in absolute terms [14], and was calculated as AFE=100 × (OR–1)/OR with 95% CIs (from the corresponding OR 95% CIs).

Thus, the percentage of all ALRI cases caused by a given virus can be calculated as overall percentage of ALRI cases positive for that virus multiplied by AFE (adjusted percentage (%a) = crude percentage (%c) × AFE), as used in previous work [15].

## RESULTS

**Figure 1:**
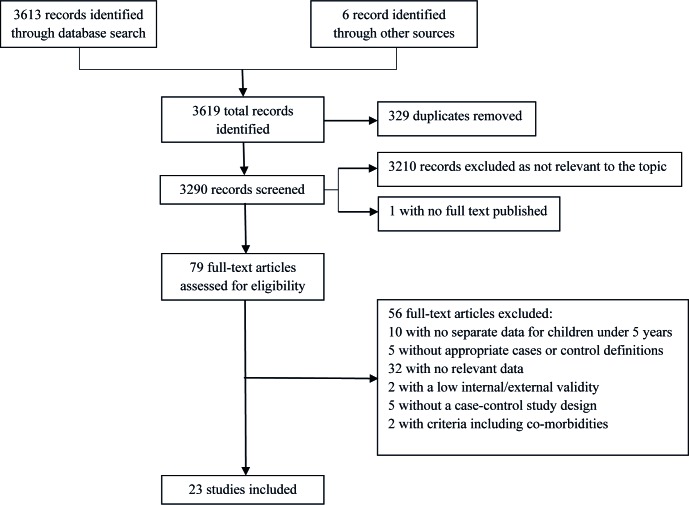
PRISMA flow diagram of the literature search.

We identified 3619 records through literature search of which only 23 studies fulfilled our strict eligibility criteria (**Figure 1**) [15–37]. Fifty–six studies were excluded for a variety of reasons including: no data specific to children under 5 years old (n=10), not fulfilling the case or control definitions (n=5), no applicable data reported for cases and controls (n=32) and other reasons (n=9). The 23 included studies were primarily conducted within developing countries (n=19) (**Table 1**). Among them, 4 unduplicated papers were from one research group in Thailand [20,22,24,37]. And most of the studies have been conducted since 2003 (n=20).

**Table 1:** Characteristics of 23 included studies

Study	Age range	Specimen(s); Diagnostic test(s)	Bacteria tested	Case group		Control Group	
**n; Ascertainment; Pro (%)**	**Definition (criteria)**	**n; Ascertainment; Sampling method**	**Definition (criteria)**
Banjul, The Gambia; Periurban; Nov 90 – Oct 92 [16]	3–59 M	NPA, LA; IIF, cell culture	Yes	119; Passive (IP); NS	P (NS)	52; Passive (H) Matched (age, area)	Healthy (No RS or malnutrition)
Baffin Island, Canada; R; Jan 02 – Mar 03 [17]	0–23 M	NPA; ELISA, DFA, m–PCR	No	121; Passive (IP); 91%	ALRI (NS)	119; Active (C); Matched (age)	Healthy (>2W no RS)
Kenya; R; Jan 07 – Dec 07 [18]	0–59 M	NPS; RT–PCR	No	726; Passive (IP); 82%	SP (WHO)	56; Passive (H); Same age group	Healthy (no RS)
Lwak and Kibera, Kenya; R; Mar 07 – Feb 11 [19]	0–59 M	NPS, OPS; qRT–PCR	Yes	538/899#; Passive (IP, OP); 36.9%	SARI (WHO)	193/109; Passive (H); Matched (age)	AS (>2W no RS)
Sa Kaeo, Thailand; R; Sep 04 – Aug 05 [20]	0–59 M	NPS; RT–PCR	No	365; Passive (IP); 50.5%*	P (CXR)	85; Passive (H); Same number in age/month	AS (>3D no RS)
Multicentre, USA; U; Nov (03 – 09) – May (03 – 09) [29]**	0–59 M	NPS; RT–PCR	No	3490; Passive (IP); NS	ARI (NS)	770; Passive (H); Same age, area, study period	Healthy (>2W no RS)
Asembo, Kenya; R; Jan 09 – Feb 10 [21]	0–59 M	NPS, OPS; sq–PCR	Yes	166/33†; Active (C), Passive (IP, OP); NS	SP (WHO)	93; Passive (H); Same age group	AS (>2W no RS)
Sa Kaeo, Thailand; R; Sep 04 – Aug 05 [22]	0–59 M	NPS; RT–PCR	No	369; Passive (IP); 51%*	P (CXR)	85; Passive (H); Same number in age/month	AS (>3D no RS)
Sa Kaeo, Thailand; R; Sep 04 – Aug 05 [37]	0–59 M	NPS; RT–PCR	No	379; Passive (IP); 45%*	ALRI (CXR)	85; Passive (H); Same number in age/month	AS (>3D no RS)
Kilifi District, Kenya; R; Jan 10 – Dec 10 [23]	1–59 M	NPS, OPS‡, IS‡; mRT–PCR	Yes	805; Passive (IP); 84%	SP (WHO)	142/227; Passive (H); Matched (age, month)	AS /URTI (No RS)
Sa Kaeo and Nakhon Phanom, Thailand; R; Jan 05 – Dec 07 [24]	0–59 M	NPS; RT–PCR	No	3809; Passive (IP); NS	ALRI (CXR)	589; Passive (H); Same age group	AS (>3D no RS)
Multicentre, USA; U; Dec 03/Oct 04 – Apr 04/Apr 05 [30]**	0–59 M	NPS; RT–PCR	No	1515; Passive (IP); 83%	ARI (NS)	790; Passive (H); Same age, area, study period	AS (>2W no RS)
Amsterdam, The Netherlands; U; Nov (07–09) – Apr (07–09) [31]**	0–23 M	NPW; mPCR	No	100; Passive (IP); NS	ARI (NS)	59; Passive (H); Same age group	AS (>1W no RS)
Quebec, Canada; U; Dec 02 – Apr 03 [32]**	0–35 M	NPA; qPCR	No	225; Passive (IP); NS	ARI (NS)	100; Passive (H); Same study period	AS (no RS)
Bhaktapur, Nepal; U; Mar 06 – Jul 07 [25]	2–35 M	NPA; mRT–PCR	No	29/671†; Passive (IP, OP); NS	P (WHO)	29/665; Active (C); Matched (age)	AS/URTI (No ab >2D)
Yuedong, China; U; Jan 07 – Dec 07 [28]	0–59 M	NPA; mRT–PCR	No	345; Passive (IP); NS	ALRI (NS)	83; Passive (H); Same age group	Healthy (>2W no RS)
Multicentre, USA; U; Dec 03/Oct 04 – Apr 04/Apr 05 [33]**	0–59 M	NPS; RT–qPCR	No	1481; Passive (IP); 82%	ARI (NS)	742; Passive (H); Same area, study period	AS (>2W no RS)
Stockholm, Sweden; U; Sep 11 – Jan 12 [34]**	0–59 M	NPA; qPCR	No	209; Passive (IP); NS	ARI (NS)	209; Passive (H); Matched (age, time)	AS (>1W no RS)
YK Delta, Alaska, USA; R; Oct 06 – Sep 07 [15]	0–35 M	NPS; sRT–PCR	No	208; Passive (IP); 60%	ALRI (NS)	381; Active (C); Same age group, unmatched	Healthy (>2W no RS)
Beersheba, Israel; U; Nov (01–05) – May (01–05) [36]**	0–59 M	NPW; RT–PCR, DIF, cell culture	No	1017; Passive (IP); 37%	P (WHO)	136; Passive (H); Same study period	Healthy (no RS)
Shantou, China; U; Jun 07 – May 08 [26]	0–24 M	NPA; mRT–PCR	No	271; Passive (IP); NS	B (NS)	82; Passive (H); NS	Healthy (no RS)
Nha Trang, Vietnam; U; Jun 08 – Aug 08 [35]**	0–59 M	NPS; mPCR	No	148; Passive (IP); 97.9%	ARI (WHO)	350; Passive (H); Same age group	Healthy (No RS, No ab >1M)
Shanghai, China; U; Oct 09 – Aug 12 [27]	0–59 M	NPA‡, NPS§; qRT–PCR	No	554; Passive (IP); NS	ALRI (CXR)	195; Passive (H); Random	Healthy (SCDC Sample Bank)

U – Urban; R – Rural, NPA – Nasopharyngeal Aspirate; NPS – Nasopharyngeal Swab; NPW – Nasopharyngeal Wash; OPS – Oropharyngeal Swab; IS – Induced Sputum; LA – Lung aspirate; IF – Immunofluorescence (IIF – Indirect; DIF – Direct); ELISA – Enzyme–linked immunosorbent assay; PCR – Polymerase chain reaction (m – multiplex; RT – reverse transcription; s – singleplex; q – quantitative/real time); (S) P – (Severe) Pneumonia; ALRI – Acute Lower Respiratory Infection; B – Bronchiolitis; NS – Not Stated; RS – Respiratory Symptoms; ab – Antibiotics; Pro – Proportion of eligible cases tested; CXR – Chest Radiography; SCDC – Shanghai Centre for Disease Control; IP – Inpatient; OP– Outpatient; H – Hospital; C – Community; D – Days; W – Weeks; M – Months; AS – Asymptomatic.

*For whole study (all ages).

†Recruitment of IP/OP.

‡Cases only.

§Controls only.

#Recruitment in the respective Lwak / Kibera site.

**There were 8 studies which were conducted for less than 12 consecutive months, and a sensitivity analysis was performed excluding these studies which found no significant differences (table in **Online Supplementary Document(Online Supplementary Document)**).

All included studies were case–control studies with an ALRI and asymptomatic/URTI groups; however some variations were still present. Of the case definitions employed, most used ALRI/ARI (n=13), while others used (severe) pneumonia (n=8), SARI (n=1) or bronchiolitis (n=1). All studies used control group which had no respiratory symptoms, of which 10 were considered otherwise “healthy”, and 2 studies [23,25] also reported URTI as control group. Of the case ascertainment used, all articles contained inpatient data. Among them, 3 studies also provided outpatient data [19,21,25]. Twenty controls were ascertained in hospital–based outpatient/clinic sites while 3 were identified in community [15,17,25].

Regarding to sampling methodology, most studies used nasopharyngeal swab (NPS) (n=10), nasopharyngeal aspirate (NPA) (n=6) and nasopharyngeal wash (NPW) (n=1) as specimen. Five studies used mixed specimens including NPA, NPS, lung aspirate and oropharyngeal swab (OPS). All studies used PCR as diagnostic testing except one study from Gambia [16], in which case indirect immunofluorescence (IIF) was applied.

Meta–analyses of virus–specific ORs were reported as well as the corresponding attributable fractions among the exposed (**Table 2**). RSV, IFV (including IFV A), PIV, MPV and RV were significantly more common in children hospitalized with ALRI than asymptomatic controls (OR (95% CI): 9.79 (4.98–19.27), 5.10 (3.19–8.14), 3.37 (1.59–7.15), 3.76 (2.45–5.78) and 1.43 (1.03–1.97), respectively). Thus, these viruses had statistically significant positive AFEs, which show clear associations between these viruses and ALRI hospitalization in young children. Therefore, this indicates the potential for substantive reductions in the number of ALRI cases were young children to be vaccinated against these viruses. In comparison, AdV, BoV and CoV were frequently detected in control children, and so did not have significantly positive AFEs. Therefore, their roles in ALRI hospitalisation were uncertain.

**Table 2:** The meta analyses of the odds ratios (OR) and attributable fraction in the exposed (AFE) of each virus and its subtype within included studies of inpatient (IP) ALRI cases relative to asymptomatic controls

Virus	Meta analyses*			Sensitivity analyses*						
Inclusion of symptomatic (URTI) controls			Inclusion of outpatient (OP) cases			
**n_s_**	**OR** (95% CI)	**AFE** (95% CI)	**n_s_**	**OR** (95% CI)	**AFE** (95% CI)	**n_s_**		**OR** (95% CI)	**AFE** (95% CI)
**RSV:**	13	**9.79** (4.98 to 19.27)	**90%** (80 to 95)	13	**8.60** (4.83 to 15.33)	**88%** (79 to 93)	14		**9.59** (5.26 to 17.49)	**90%** (81 to 94)
A	1	–	–	1	–	–	1	–		–
B	1	–	–	1	–	–	1	–		–
**IFV:**	10	**5.10** (3.19 to 8.14)	**80%** (69 to 88)	10	**3.39** (1.64 to 7.02)	**71%** (39 to 86)	10	**5.54** (3.56 to 8.62)		**82%** (72 to 88)
A	8	**5.97** (3.29 to 10.81)	**83%** (70 to 91)	8	**3.99** (1.68 to 9.49)	**75%** (40 to 89)	9	**6.07** (3.35 to 10.98)		**84%** (70 to 91)
B	9	**2.70** (0.97 to 7.53)	**63%** (–3 to 87)	9	**2.70** (0.97 to 7.54)	**63%** (–3 to 87)	9	**3.36** (1.48 to 7.65)		**70%** (32 to 87)
C	1	–	–	1	–	–	1	–		–
**PIV:**	11	**3.37** (1.59 to 7.15)	**70%** (37 to 86)	11	**3.14** (1.25 to 7.85)	**68%** (20 to 87)	11	**4.07** (1.91 to 8.67)		**75%** (48 to 88)
1	6	**2.52** (0.79 to 8.07)	**60%** (–27 to 88)	6	**2.10** (0.64 to 6.88)	**52%** (–56 to 85)	6	**3.10** (1.21 to 7.94)		**68%** (17 to 87)
2	7	**2.22** (0.80 to 6.16)	**55%** (–25 to 84)	7	**1.18** (0.33 to 4.27)	**15%** (–203 to 77)	7	**2.34** (0.94 to 5.83)		**57%** (–6 to 83)
3	8	**2.19** (0.97 to 4.97)	**54%** (–3 to 80)	8	**2.05** (0.77 to 5.43)	**51%** (–30 to 82)	8	**4.14** (1.24 to 13.82)		**76%** (19 to 93)
4	1	–	–	1	–	–	1	–		–
**MPV**	10	**3.76** (2.45 to 5.78)	**73%** (59 to 83)	10	**3.61** (2.38 to 5.46)	**72%** (58 to 82)	11	**3.84** (2.51 to 5.88)		**74%** (60 to 83)
**AdV**	10	**1.13** (0.71 to 1.80)	**12%** (–41 to 44)	10	**1.16** (0.76 to 1.77)	**14%** (–32 to 44)	10	**1.13** (0.71 to 1.80)		**12%** (–41 to 44)
**RV**	11	**1.43** (1.03 to 1.97)†	**30%** (3 to 49)	11	**1.41** (1.03 to 1.93)	**29%** (3 to 48)	11	**1.43** (1.03 to 1.97)		**30%** (3 to 49)
**BoV**	8	**1.20** (0.36 to 3.98)	**17%** (–178 to 75)	8	**1.20** (0.36 to 3.98)	**17%** (–178 to 75)	8	**1.20** (0.36 to 3.98)		**17%** (–178 to 75)
**CoV**	8	**1.03** (0.80 to 1.33)	**3%** (–25 to 25)	8	**0.94** (0.74 to 1.19)	**–6%** (–35 to 16)	8	**1.03** (0.80 to 1.33)		**3%** (–25 to 25)
**HKU1**	4	**0.61** (0.34 to 1.09)	**–64%** (–194 to 8)	4	**0.61** (0.34 to 1.09)	**–64%** (–194 to 8)	4	**0.61** (0.34 to 1.09)		**–64%** (–194 to 8)
**NL63**	5	**0.68** (0.38 to 1.24)	**–47%** (–163 to 19)	5	**0.71** (0.41 to 1.25)	**–41%** (–144 to 20)	5	**0.68** (0.38 to 1.24)		**–47%** (–163 to 19)
**229E**	4	**1.47** (0.58 to 3.72)	**32%** (–72 to 73)	4	**1.42** (0.46 to 4.43)	**30%** (–117 to 77)	4	**1.47** (0.58 to 3.72)		**32%** (–72 to 73)
**OC43**	5	**0.91** (0.32 to 2.64)	**–10%** (–213 to 62)	5	**0.84** (0.39 to 1.80)	**–19%** (–156 to 44)	5	**0.91** (0.32 to 2.64)		**–10%** (–218 to 62)

ns ***–*** Number of studies; N/A – Not applicable; 95% CI – 95% confidence interval; RSV – Respiratory syncytial virus; IFV – Influenza; PIV – Parainfluenza; MPV – Human metapneumovirus; AdV – Adenovirus; RV – Rhinovirus; BoV – Bocavirus; CoV – Coronavirus; OR – Odds ratio; AFE – Attributable fraction among the exposed.

*From the random–effects model.

†OR=1.40 (1.02 to 1.92) and AFE=28% (2 to 48) when studies testing all other enterovirus are excluded.

Sensitivity analyses were also performed to investigate the effect of inclusion of symptomatic (URTI) controls, and of outpatient ALRI cases (**Table 2**). Data on inclusion of outpatient cases had little impact on the associations observed. However, this does not necessarily indicate similarity between the association of inpatient and outpatient ALRI patients. In comparison, the inclusion of symptomatic controls had a more substantial influence, which reduced the strength of association with every virus, except AdV and CoV–NL63.

## DISCUSSION

This is the first systematic review to evaluate and summarise the literature surrounding the viral aetiology of ALRI in young children. Our aim was to summarise good quality data on the absolute effects of the viral exposure and hence to inform causal inference in ALRI aetiological studies which report respiratory viral data. Our review summarises data from 18 592 cases of ALRI in young children reported across 23 studies. We demonstrated stronger evidence (defined here as a statistically significant OR >3) in support of a causal attribution when a virus is identified in young children presenting with ALRI for RSV (OR 9.59–9.79; AFE 90%), IFV (OR 5.10–5.54; AFE 80%), PIV (OR 3.37–4.07; AFE 70%) and MPV (OR 3.76–3.84; AFE 73%). There was less strong evidence (defined here as a statistically significant OR 1–3) for RV (OR 1.43; AFE 30%). There was no statistically significant difference between viral identification in ALRI cases and controls for the other respiratory viruses studied: AdV, BoV, CoV.

These findings should inform the results of studies which seek to estimate the global / regional / national burden of disease due to these viruses. They support the role of RSV, IFV, PIV and MPV as important causes of ALRI in young children (although disease burden estimates should take into account the AFE estimates that we report – thus the true global burden of RSV/IFV/PIV/MPV pneumonia may be 90%/80%/70%/73% of the values reported in recent publications). Applying these estimates to the burden of severe (hospitalised) pneumonia in 2010 [38], we estimate that the likely true burden of RSV and influenza associated ALRI for that year would be about 2.9 (95% CI 1.5–5.5) million and 0.8 (0.3–2.2) million respectively. There is considerable international attention on RSV and IFV pneumonia in young children at this time when novel vaccine strategies are being evaluated and prioritised and more accurate disease burden estimates (using these results) would help inform future policies and interventions.

Several methodological issues could affect our results: case ascertainment, case definition, clinical specimen and confounding. Twenty–two of the 23 studies used passive hospital–based case ascertainment. Several previous studies have shown that children in developing countries, particularly those residing in rural areas, have in general, limited access to healthcare [39], and health care seeking behaviour is often delayed or absent [40–42]. This potentially introduced a selection bias. Similarly, only three studies used community based controls [15,19,25]. Hospital ascertained controls may not reflect the general population, and may have other health conditions potentially affecting their viral carriage, especially those with URTI. The ideal control group for these studies would be a random sample of an age and sex matched child population from the same area of residence studied at the same time. Studies, however, recruited controls who were either selected as healthy (asymptomatic) and so biased in favour of those not exposed to the respiratory virus (yielding a falsely high OR) or those who were selected to have respiratory symptoms and so biased in favour of those who had been exposed to the respiratory virus (yielding a falsely low OR). Consistent with this interpretation, we found (**Table 2**) odds ratios (of ALRI given viral identification) to be consistently greater where the control group were “healthy” and asymptomatic rather than symptomatic (URTI). We consider that the value of the OR based on a population–based control group as described above would lie between these two values.

Seven of the included studies [17,20–23,25,36] employed the WHO case definition for pneumonia [43] and this standardised approach enhanced the comparability of results between these studies. These criteria have high sensitivity for pneumonia [44], but lower specificity with overlap with other conditions [45], particularly malaria [46] and wheezing disorders. This tends to inflate the number of “cases” and may contribute to an apparent low level of detection of pathogenic viruses.

All included studies obtained upper respiratory tract specimens (i.e. described as nasopharyngeal secretions, nasopharyngeal wash samples, nasopharyngeal aspirate samples, oropharyngeal samples). Although their differing sensitivities could result in some heterogeneity [47,48], they are broadly comparable and have common flaws. As viruses identified could be from a coincidental URTI in ALRI cases, the sole use of these specimens can only provide supportive evidence for causality. Lung aspiration is considered the gold–standard sampling technique given it is directly obtained from the infection site [49], which would indicate aetiological significance in ALRI. However, its invasive nature and rate of complications limit its use.

Several potential confounding factors could have distorted the observed associations. Only three studies calculated appropriately adjusted ORs to account for confounding effects from age [24], or age and season [21,23]. Instead, matching of cases and controls was more commonly used – performed by age [16,18,19,25], or age and month [23,34]. Nevertheless, despite the use of matching, no studies maintained this pairing to allow OR meta–estimate calculation. While all studies were conducted on young children (under 5 years), six [15,17,25,26,31,32] were further restricted. As age is an ALRI risk factor [50], this could potentially affect the viral profile detected, introducing further heterogeneity. However, no enough data were provided to estimate the strength of association in narrower age bands.

In addition, multiple aetiological agents may often be identified in young children with ALRI, making the individual contribution of each agent difficult to define. Many of the included studies did not provide virological data that excludes coinfections, so viruses detected in these cases could conceivably fulfil any etiological role. The high sensitivity of polymerase chain reaction (PCR) is important for accurate assessment of aetiological contribution. However, the high rates of viral co–infection detection may overstate the individual contribution [8].

Furthermore, any viruses detected could be from a nascent infection or persistent from a previous infection [51]. These could explain more ‘pathogenic’ viruses (such as RSV) being identified in asymptomatic children. Some viruses are detectable for weeks before and after ALRI, [52–54] and so the studies only assessing asymptomatic status without considering past or future history may yield false positive findings [16,17,23,32,36].

Moreover, the small sample size [16,25,31], undoubtedly contributed to the imprecise 95% CIs in **Table 2**. This may have also led to the non–detection of statistically significant ALRI–virus associations as in some case and/or control groups. Two studies [15,17] included children of Inuit ancestry from USA or Canada and were found to have high ALRI incidence. The viral associations observed in these two studies may not be generalizable to other populations.

The use of the AFE allows quantification of the excess percentage of ALRI cases due to exposure in absolute terms [55]. However, it assumes the observed association between the virus (and/or related factors) and ALRI is causal [14], and, in practice, this will undoubtedly have led to extreme estimations. Furthermore, strict interpretation would entail construal of negative values as indicative of the percentage of ALRI prevented by viral exposure [56], which is biologically implausible.

A virus (or any pathogen) can be considered to be associated with ALRI when detected with a significantly higher frequency in cases than controls without respiratory symptom (asymptomatic). However, coincidence, while necessary, is insufficient for proof of a causal role between a virus and ALRI [57]. Other alternative explanations must first be refuted before causality can be concluded [58].

Firstly, the virus could be an “innocent bystander” which is more prevalent in patients with ALRI, but has no causal role. Such an effect may be observed due to immunocompromised status from the true causal infection, or nosocomial infections. Secondly, the virus may be a risk factor for ALRI development, but not itself the primary cause. It has been well established that viral infections predispose to subsequent bacterial infection, although the exact mechanisms are still debated [59,60]. Indeed, influenza and RSV epidemics are commonly observed to precede those of bacterial pneumonia [61–63]. Thirdly, the virus may be necessary to cause ALRI, but is not sufficient to do so without the concurrent presence of one or more other causal factors. There are numerous risk factors that have been associated with ALRI, both host and environment [50]. Furthermore, there has been lethal synergism observed in viral–bacterial superinfections [64,65]. These, singularly or in combination, may provide the opportunity for the respiratory virus to cause ALRI. Fourthly, the virus may be the direct and sole cause of ALRI, with causality yet to be confirmed. Finally, the virus may be the joint cause of ALRI along with other concurrent viral respiratory infections.

Another essential criterion required for the determination of causality is establishment of the temporal sequence of exposure and outcome [57]. As exposure is investigated after the outcome in case–control studies, these cannot provide this evidence. Therefore, there is a rationale for the conduct of birth cohort studies with routine surveillance of children to track the circulation and course of respiratory viral infections. “Vaccine–probe studies” could be used to gain experimental evidence of each virus as a causal pathogen [66], although this is limited by vaccine availability of effective vaccines. These would allow conclusive assessment of the burden of ALRI attributable to each virus. Considerations of the causal role of these viruses are further complicated by the fact that a recent respiratory virus infection may have caused temporary immune–suppression leading to a subsequent viral or bacterial infection even though the initial infection can no longer be detected. Influenza viral infections leading to subsequent pneumococcal or staphylococcal respiratory infections have been well described [67]. This may result in an under–estimation of the burden of disease associated with respiratory viral infection.

Notwithstanding these limitations, this review provides clear evidence in favour of the causal role of RSV, IFV, PIV, MPV and to a lesser extent RV in childhood ALRI and presents first estimate of the proportion of ALRI cases that can be attributed to the viral exposure. Aetiological studies which simply report rates of viral identification as causal should make attempt to interpret findings in terms of the proportion of ALRI cases among children in whom a respiratory virus is identified that can be attributed to this viral exposure.
