# Supplementary material for: Aetiological role of common respiratory viruses in acute lower respiratory infections in children under five years: A systematic review and meta–analysis
Source: J Glob Health. 2015 Jun 17;5(1):010408. doi: 10.7189/jogh.05.010408 (PMC4593292; doi:10.7189/jogh.05.010408)
Supplement: Online Supplementary Document [file jogh-05-010408-s001.pdf]

Online Supplementary Document

Shia et al. The etiological role of common respiratory viruses in acute lower respiratory infections in children under five years: A systematic review and meta-analysis

J Glob Health 2015;5:010408

Appendix: Search strategy

Medline
1. Prospective study.mp. or exp Prospective Studies/
2. control*.mp.
3. exp Case-Control Studies/ or case control.mp.
4. (case* adj2 control*).mp. [mp=title, abstract, original title, name of substance word, subject heading word, keyword heading word, protocol supplementary concept word, rare disease supplementary concept word, unique identifier]
5. virus.mp. or exp Viruses/
6. exp Virus Diseases/ or virus disease*.mp.
7. pneumonia.mp. or exp Pneumonia/ or exp Pneumonia, Viral/
8. bronchiolitis.mp. or exp Bronchiolitis/ or exp Bronchiolitis, Viral/
9. exp Respiratory Tract Infections/ or respiratory infection*.mp.
10. exp Respiratory Tract Diseases/ or respiratory disease*.mp.
11. 1 and 2
12. 3 or 4 or 11
13. 5 or 6
14. 7 or 8 or 9 or 10
15. 12 and 13 and 14
16. limit 15 to (yr="1990 -Current" and ("all infant (birth to 23 months)" or "newborn infant (birth to 1 month)" or "infant (1 to 23 months)" or "preschool child (2 to 5 years)") and (female or humans or male))

Embase
1. virus*.mp. or exp virus/
2. exp virus infection/ or virus disease*.mp.
3. exp community acquired pneumonia/ or exp pneumonia/ or exp virus pneumonia/ or exp infectious pneumonia/ or pneumonia.mp.
4. bronchiolitis.mp. or exp bronchiolitis/ or exp viral bronchiolitis/
5. exp respiratory tract infection/ or exp lower respiratory tract infection/ or respiratory infection*.mp.
6. exp respiratory tract disease/ or respiratory disease*.mp.
7. exp case control study/ or case control.mp.
8. (case* adj2 control*).mp. [mp=title, abstract, subject headings, heading word, drug trade name, original title, device manufacturer, drug manufacturer, device trade name, keyword]
9. prospective study.mp. or exp prospective study/
10. control*.mp. or exp control group/ or exp control/
11. 1 or 2
12. 3 or 4 or 5 or 6
13. 9 and 10
14. 7 or 8 or 13
15. 11 and 12 and 14
16. limit 15 to (yr="1990 -Current" and (infant or child or preschool child <1 to 6 years>))

Global Health
1. virus.mp. or exp viruses/
2. viral disease*.mp. or viral diseases.sh.
3. exp community acquired pneumonia/ or pneumonia.mp. or exp pneumonia/
4. bronchiolitis.mp. or exp bronchiolitis/

5. respiratory infection*.mp.
6. respiratory disease*.mp. or respiratory diseases.sh.
7. case control.mp.
8. exp case-control studies/
9. (case* adj2 control*).mp. [mp=abstract, title, original title, broad terms, heading words]
10. prospective study.mp.
11. exp control/ or control*.mp.
12. 1 or 2
13. 3 or 4 or 5 or 6
14. 10 and 11
15. 7 or 8 or 9 or 14
16. 12 and 13 and 15
17. limit 16 to yr="1990 -Current"

LILACS
tw:(virus OR viral) AND tw:(pneumonia OR bronchiolitis OR alri OR lrti OR "lower respiratory" OR "respiratory infection") AND tw:(control)

CNKI
Topic: respiratory infection or pneumonia (vague)
Or Topic: bronchiolitis (vague)
And Topic: virus (vague)
And Topic: case control (vague)
And Topic: child (vague)
Publication time: 1st Jan 1990 – 19th Mar 2014

Wanfang Data
All (vague): respiratory infection or
All (vague): pneumonia or
All (vague): bronchiolitis and
All (vague): virus and
All (vague): case control and
All (vague): child
Time: 1990 – 2014

Chongqing VIP
Title/key word: respiratory infection
Or Title/key word: pneumonia
And Title/key word: virus
And Title/key word: case control
And Title/key word: child
Time: 1990 – 2014

Appendix 1: The meta analyses of the odds ratios (OR) and attributable fraction in the exposed (AFE) of each virus and subtype within included studies of inpatient (IP) ALRI cases relative to asymptomatic controls (after excluding the articles with less than 1 year study period).

**Virus**	**Meta Analyses**			**Sensitivity Analyses**					
				**Inclusion of symptomatic (URTI) controls**			**Inclusion of outpatient (OP) cases**		
	**n_s**	**OR (95% CI)**	**AFE (95% CI)**	**n_s**	**OR (95% CI)**	**AFE (95% CI)**	**n_s**	**OR (95% CI)**	**AFE (95% CI)**
**RSV**	10	**8.37** (3.92 to 17.91)	**88%** (74 to 94)	10	**7.14** (3.84 to 13.29)	**86%** (74% to 92%)	11	**8.40** (4.34 to 16.25)	**88%** (77 to 94)
**A**	1	-	-	1	-	-	1	-	-
**B**	1	-	-	1	-	-	1	-	-
**IFV**	8	**5.43** (3.14 to 9.38)	**82%** (68 to 89)	8	**3.45** (1.45 to 8.18)	**71%** (31 to 88)	8	**6.00** (3.61 to 9.96)	**83%** (72 to 90)
**A**	6	**5.88** (2.83 to 12.22)	**83%** (65 to 92)	6	**3.68** (1.16 to 11.68)	**73%** (14 to 91)	7	**6.04** (2.91 to 12.52)	**83%** (66 to 92)
**B**	7	**2.76** (0.91 to 8.33)	**64%** (-10 to 88)	7	**2.76** (0.91 to 8.34)	**64%** (-10 to 88)	7	**3.48** (1.48 to 8.23)	**71%** (32 to 88)
**C**	1	-	-	1	-	-	1	-	-
**PIV**	8	**2.90** (1.30 to 6.45)	**66%** (23 to 84)	8	**2.61** (1.00 to 6.84)	**62%** (0 to 85)	8	**3.61** (1.61 to 8.07)	**72%** (38 to 88)
**1**	5	**1.87** (0.50 to 7.01)	**47%** (-100 to 86)	5	**1.56** (0.44 to 5.58)	**36%** (-127 to 82)	5	**2.59** (0.91 to 7.36)	**61%** (-10 to 86)
**2**	5	**2.22** (0.80 to 6.16)	**55%** (-25 to 84)	5	**1.27** (0.27 to 5.86)	**21%** (-270 to 83)	5	**2.34** (0.94 to 5.83)	**57%** (-6 to 83)
**3**	6	**2.42** (0.90 to 6.49)	**59%** (-11 to 85)	6	**2.18** (0.73 to 6.52)	**54%** (-37 to 85)	6	**4.19** (1.17 to 14.94)	**76%** (15 to 93)
**4**	1	-	-	1	-	-	1	-	-
**MPV**	6	**3.24** (1.18 to 8.88)	**69%** (15 to 89)	6	**2.93** (1.26 to 6.81)	**66%** (21 to 85)	7	**3.69** (1.53 to 8.92)	**73%** (35 to 89)
**AdV**	7	**0.95** (0.56 to 1.63)	**-5%** (-79 to 39)	7	**1.00** (0.61 to 1.63)	**0%** (-64 to 39)	7	**0.96** (0.56 to 1.64)	**-4%** (-79 to 39)
**RV**	7	**1.36** (0.92 to 2.02)ł	**26%** (-9 to 50)	7	**1.34** (0.92 to 1.93)	**25%** (-8 to 48)	7	**1.36** (0.92 to 2.01)	**26%** (-9 to 50)
**BoV**	4	**1.51** (0.24 to 9.63)	**34%** (-317 to 90)	4	**1.51** (0.24 to 9.63)	**34%** (-317 to 90)	4	**1.51** (0.24 to 9.63)	**34%** (-317 to 90)
**CoV**	4	**1.09** (0.68 to 1.76)	**8%** (-47 to 43)	4	**0.83** (0.57 to 1.22)	**-20%** (-75 to 18)	4	**1.09** (0.68 to 1.76)	**8%** (-47 to 43)
**HKU1**	2	**0.08** (0.00 to 1.24)	**-1150%** (-EV to 19)	2	**0.08** (0.00 to 1.24)	**-1150%** (-EV to 19)	2	**0.08** (0.00 to 1.24)	**-1150%** (-EV to 19)
**NL63**	3	**0.23** (0.01 to 3.78)	**-335%** (-9900 to 74)	3	**0.68** (0.17 to 2.83)	**-47%** (-488 to 65)	3	**0.23** (0.01 to 3.78)	**-335%** (-9900 to 74)
**229E**	3	**1.14** (0.34 to 3.80)	**12%** (-194 to 74)	3	**1.10** (0.26 to 4.67)	**9%** (-285 to 79)	3	**1.14** (0.34 to 3.80)	**12%** (-194 to 74)
**OC43**	3	**0.65** (0.10 to 4.27)	**-54%** (-900 to 77)	3	**0.54** (0.16 to 1.84)	**-85%** (-525 to 46)	3	**0.65** (0.10 to 4.27)	**-54%** (-900 to 77)

Abbreviations: n_s = Number of studies; N/A = Not applicable; 95% CI = 95% Confidence Interval; EV = Extreme value; RSV = Respiratory syncytial virus; IFV = Influenza; PIV = Parainfluenza; MPV = human metapneumovirus; AdV = Adenovirus; RV = Rhinovirus; BoV = Bocavirus; CoV = Coronavirus; OR = Odds ratio; AFE = Attributable fraction among the exposed.

***** From the random-effects model. ł The OR = 1.26 (0.95 to 1.67) and AFE = 21% (-5 to 40) when studies testing all enterovirus are excluded.
